# A novel risk score for the prediction of airway management in patients with deep neck space abscess: a multicenter retrospective cohort study

**DOI:** 10.1186/s40560-021-00554-8

**Published:** 2021-05-20

**Authors:** Yu Lin, Wenxiang Gao, Huijun Yue, Weixiong Chen, Tianrun Liu, Jin Ye, Qian Cai, Fei Ye, Long He, Xingqiang Xie, Guoping Xiong, Bin Wang, Feng Pang, Pei Li, Jianhui Wu, Bin Wang, Junru Huang, Weiping Wen, Wenbin Lei

**Affiliations:** 1 Otorhinolaryngology Hospital, The First Affiliated Hospital, Sun Yat-Sen University, No. 58 Zhongshan 2nd Road, Guangzhou, Guangdong People’s Republic of China; 2 Department of Otorhinolaryngology-Head and Neck Surgery, First People’s Hospital of Foshan, Foshan, Guangdong People’s Republic of China; 3 Department of Otorhinolaryngology-Head and Neck Surgery, Sixth Affiliated Hospital of Sun Yat-Sen University, Guangzhou, Guangdong People’s Republic of China; 4 Department of Otorhinolaryngology-Head and Neck Surgery, Third Affiliated Hospital of Sun Yat-Sen University, Guangzhou, Guangdong People’s Republic of China; 5 Department of Otorhinolaryngology-Head and Neck Surgery, Sun Yat-Sen Memorial Hospital of Sun Yat-Sen University, Guangzhou, Guangdong People’s Republic of China; 6 Department of Otorhinolaryngology-Head and Neck Surgery, Zhongshan People’s Hospital, Zhongshan, Guangdong People’s Republic of China; 7 Department of Otorhinolaryngology-Head and Neck Surgery, First People’s Hospital of Guangzhou, Guangzhou, Guangdong People’s Republic of China; 8 Department of Otorhinolaryngology-Head and Neck Surgery, First People’s Hospital of Zhaoqing, Zhaoqing, Guangdong People’s Republic of China; 9 Department of Otorhinolaryngology-Head and Neck Surgery, Jiangmen Central Hospital Affiliated Jiangmen Hospital of Sun Yat-Sen University, Jiangmen, Guangdong People’s Republic of China

**Keywords:** Deep neck space abscess, Risk score, Airway management, Multicenter study

## Abstract

**Background:**

Airway management, including noninvasive endotracheal intubation or invasive tracheostomy, is an essential treatment strategy for patients with deep neck space abscess (DNSA) to reverse acute hypoxia, which aids in avoiding acute cerebral hypoxia and cardiac arrest. This study aimed to develop and validate a novel risk score to predict the need for airway management in patients with DNSA.

**Methods:**

Patients with DNSA admitted to 9 hospitals in Guangdong Province between January 1, 2015, and December 31, 2020, were included. The cohort was divided into the training and validation cohorts. The risk score was developed using the least absolute shrinkage and selection operator (LASSO) and logistic regression models in the training cohort. The external validity and diagnostic ability were assessed in the validation cohort.

**Results:**

A total of 440 DNSA patients were included, of which 363 (60 required airway management) entered into the training cohort and 77 (13 required airway management) entered into the validation cohort. The risk score included 7 independent predictors (p < 0.05): multispace involvement (odd ratio [OR] 6.42, 95% confidence interval [CI] 1.79–23.07, p < 0.001), gas formation (OR 4.95, 95% CI 2.04–12.00, p < 0.001), dyspnea (OR 10.35, 95% CI 3.47–30.89, p < 0.001), primary region of infection, neutrophil percentage (OR 1.10, 95% CI 1.02–1.18, p = 0.015), platelet count to lymphocyte count ratio (OR 1.01, 95% CI 1.00–1.01, p = 0.010), and albumin level (OR 0.86, 95% CI 0.80–0.92, p < 0.001). Internal validation showed good discrimination, with an area under the curve (AUC) of 0.951 (95% CI 0.924–0.971), and good calibration (Hosmer–Lemeshow [HL] test, p = 0.821). Application of the clinical risk score in the validation cohort also revealed good discrimination (AUC 0.947, 95% CI 0.871–0.985) and calibration (HL test, p = 0.618). Decision curve analyses in both cohorts demonstrated that patients could benefit from this risk score. The score has been transformed into an online calculator that is freely available to the public.

**Conclusions:**

The risk score may help predict a patient’s risk of requiring airway management, thus advancing patient safety and supporting appropriate treatment.

## Introduction

Deep neck space abscess (DNSA) is a rare infectious disease with an incidence of 0.09–0.15‰, but it is still a globally recognized critical public health disease [1]. Due to mucosal edema and abscess compression in the airway, patients with DNSA frequently have airway compromise, which can lead to airway loss and result in acute hypoxia. Airway management is an essential part of the treatment strategy for DNSA to reverse acute hypoxia, which aids in avoiding acute cerebral hypoxia and cardiac arrest [2]. Thus, the early detection of patients who are likely in need of airway management is critical.

According to previous studies, the proportion of patients with DNSA who need airway management is 16.8–38.2%, while the proportion of patients with dyspnea at the time of presentation at the hospital is only 5.6–12.8% [3–5]. The difference in these proportions suggests that patients with DNSA often exhibit rapid worsening of the infection and is difficult to assess their dynamic condition. In particular, evaluating whether airway management is needed in patients with compromised airways at the time of presentation at the hospital is very challenging. Moreover, the procedures involved in airway management in patients with compromised airways are urgent and complex, requiring the close cooperation of clinicians from multiple disciplines. The early identification of patients who are likely to need airway management facilitates the efficient establishment of a multidisciplinary team. A predication tool that could indicate the need to prepare for airway management would help resolve these issues.

Prediction models of airway management have been reported in many studies [6–8]. Although there are clinical prediction models for drainage surgery, complications, and prognosis in patients with DNSA [9–11], a prediction model for airway management in DNSA has not been reported to date.

Therefore, we aimed to fit the diverse clinical data from multiple hospitals and construct a clinical risk score based on the DNSA cohort in the Pearl River Delta of Guangdong Province to help identify patients at the time of presentation at the hospital who are likely to require airway management and to assess the diagnostic abilities and clinical benefits of this risk score.

## Materials and methods

### Study population

This is a multicenter study from 9 hospitals (the First Affiliated Hospital of Sun Yat-Sen University, the First People’s Hospital of Foshan, the Sixth Affiliated Hospital of Sun Yat-Sen University, the Third Affiliated Hospital of Sun Yat-Sen University, Sun Yat-Sen Memorial Hospital of Sun Yat-Sen University, Zhongshan People’s Hospital, the First People’s Hospital of Guangzhou, the First People’s Hospital of Zhaoqing, and Jiangmen Central Hospital Affiliated Jiangmen Hospital of Sun Yat-Sen University) in five different cities (Guangzhou, Foshan, Zhaoqing, Zhongshan, and Jiangmen) in Guangdong Province, and it represents the clinical characteristics of patients with DNSA in the Pearl River Delta of Guangdong Province, China. Data were retrospectively extracted from electronic medical records with a standard Excel form in these nine hospitals between January 1, 2015, and December 31, 2020, and the data included disease history, symptoms, signs, imaging characteristics, and laboratory test results. This study was approved by each institutional Ethics Committee for Research and Publication. Written informed consent was waived owing to the use of deidentified retrospective data.

DNSAs (retropharyngeal, parapharyngeal, and submandibular abscesses) were identified using the diagnosis codes (J39.002, J39.004, and L02.051) of the International Classification of Diseases, Tenth Revision, Clinical Modifications (ICD-10-CM) [12]. Patients with the following conditions were excluded: (1) had benign or malignant tumors, (2) had a history of chemoradiotherapy, (3) had infections secondary to surgical neck trauma, and (4) did not accept treatment. Each record was checked independently by 2 clinicians. Patients from the above 9 hospitals between January 1, 2015, and December 31, 2019, were entered into the training cohort. From January 1, 2020, to December 31, 2020, a validation cohort from 6 hospitals (The First Affiliated Hospital of Sun Yat-Sen University, The First People’s Hospital of Foshan, The First People’s Hospital of Zhaoqing, The Third Affiliated Hospital of Sun Yat-Sen University, Zhongshan People’s Hospital, and Jiangmen Central Hospital Affiliated Jiangmen Hospital of Sun Yat-Sen University) was established consecutively with the same standards as the training cohort.

### Data collection

Data were collected by 2 experienced clinicians. Potential predictive variables included the following patient characteristics at the hospital visit: demographic predictors, medical history, clinical signs and symptoms, imaging results, and laboratory findings. Imaging and laboratory examinations were performed immediately after admission, and the results were available within 30 min. Demographic predictors collected for the study included age (> 18 years), sex, smoking, alcoholism, and disease duration (from the time of symptom onset to the time of hospital visit). Medical history included diabetes mellitus, hypertension, and antibiotic allergy. Clinical signs and symptoms included pharyngalgia, odynophagia, neck pain, neck swelling, dysphagia, trismus, hoarseness, dyspnea, body temperature, and heart rate. Imaging results included primary regions of infection, gas formation, and multispace involvement. Primary regions of infection were divided into three variables (suprahyoid, infrahyoid, and retropharyngeal regions) based on the review of Vieira et al. [13]. The infrahyoid region was identified as a protective factor in the univariate analysis, while the suprahyoid and retropharyngeal regions were identified as risk factors. As a result, the infrahyoid region was regarded as a reference variable for suprahyoid and retropharyngeal regions in the multivariate analysis. Laboratory findings, including lymphocyte counts, the percentage of neutrophils (NEUT%), platelet counts, platelet to lymphocyte ratio (PLR), albumin, red blood cell (RBC) count, hemoglobin (Hb), and blood glucose, have been reported to be associated with poor condition in DNSA [10, 14–18]. The other potential predictors were not considered if missing values were more than 5%. All categorical variables were defined as yes or no in the data analysis except for primary region of infection.

### Outcomes

The extent of airway compromise was immediately assessed when each patient presented at the hospital. The criteria for the need for airway management in this study were as follows: (1) symptoms of third- or fourth-degree laryngeal obstruction due to mucosal edema and abscess compression, (2) oxyhemoglobin saturation less than 90%, and (3) excessive secretions that were difficult to expectorate due to severe neck pain or airway compromise. Emergency or preventive tracheostomy was performed in patients with emergency airway loss or severe pneumonia. Patients with successfully endotracheal intubation that had been performed due to respiratory distress were admitted to the intensive care unit (ICU) for ventilation. Therefore, the outcome of this study was airway management, which was defined as follows: (1) tracheotomy and (2) endotracheal intubation. Patients who received airway management due to the performance of surgical drainage under general anesthesia and were extubated immediately or within 24 h were not included in the intervention group. Those who had undergone endotracheal intubation or tracheostomy before being referred to the study hospitals were excluded. The procedure for airway management was completed by the multidisciplinary cooperation of otolaryngologists, ICU physicians, emergency department (ED) physicians, and anesthesiologists. The procedure followed the Practice Guidelines for Management of the Difficult Airway, which was updated by the American Society of Anesthesiologists (ASA) in 2013 [19].

### Statistical analysis

Continuous variables are presented as the medians and interquartile ranges (IQRs), while categorical variables are presented as frequencies and percentages (%). Univariate analyses were performed with Mann–Whitney *U* tests, chi-square tests, and Fisher’s exact tests. Least absolute shrinkage and selection operator (LASSO) regression was applied to minimize the potential collinearity of variables measured from the same patient. A 10-fold cross-validation method was adopted to avoid overfitting of the model. Tuning parameter λ of minimum 1 standard error to the minimum was determined with cross-validation. Variables selected in LASSO regression were further analyzed in a logistic regression model, and a clinical risk score of the nomogram was built in the training cohort to visualize the logistic regression model. The odds ratio (OR) and 95% confidence interval (CI) were calculated by the model. Finally, a web-based risk calculator (https://7-414-5-19.shinyapps.io/ClinicalRiskScore/) was constructed according to the risk score.

To assess the discriminative performance of the risk score, the area under the curve (AUC) of the receiver operating characteristic (ROC) curve was measured. A calibration curve was generated for the evaluation of calibration, combined with the Hosmer–Lemeshow (HL) test. An insignificant HL test statistic implies good calibration. Moreover, decision curve analysis (DCA) was carried out to assess the clinical usefulness of the generated risk score by evaluating net benefits at various threshold probabilities in the training cohort. Then, the risk score built in the training cohort was further validated in the validation cohort. The performance of the risk score in terms of discrimination, calibration, and DCA was assessed in the validation cohort using the same methods described above. A *p*-value less than 0.05 was considered significant in each statistical analysis. Statistical analyses were performed using Statistical Product and Service Solutions (SPSS) version 25.0 software (IBM Corp., Armonk, NY, USA) and the R environment, version 4.0.2 (R Foundation for Statistical Computing, Vienna, Austria).

## Results

### Characteristics of the training cohort

**Fig. 1:**
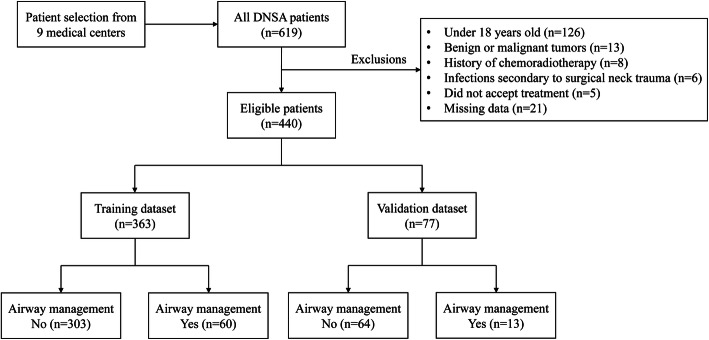
Flowchart outlining patient selection and grouping process. DNSA, deep neck space abscess

The overall flowchart of patient selection is shown in Fig. 1. In the training cohort, we collected data from 363 patients (246 males and 117 females) from 9 hospitals in the Pearl River Delta of Guangdong Province between January 1, 2015, and December 31, 2019. The median age of patients in the cohort was 51 years (IQR 37–61 years). Diabetes mellitus and hypertension were identified in 94 (25.9%) and 51 (14.0%) patients, respectively. The most common primary infection site was in the suprahyoid region, followed by infrahyoid and retropharyngeal regions. Enhanced CT confirmed that 168 patients (46.3%) had multispace involvement, and 99 patients (27.3%) had gas formation. The other clinical characteristics of the training cohort are presented in Table 1**.**

**Table 1:** Demographics and clinical characteristics of patients with DNSAs in the training cohort

Predictors	All (n = 363)	Airway management		p
		Yes (n = 60)	No (n = 303)	
Age, median (IQR), years	51.0 (37.0–61.0)	61.5 (54.0–67.0)	47.0 (35.0–58.0)	**< 0.001**
Sex, male, n (%)	246 (67.8)	41 (68.3)	205 (67.7)	0.918
Antibiotic allergy, n (%)	29 (8.0)	5 (8.3)	24 (7.9)	0.915
Disease duration, median (IQR), days	7.0 (4.0–11.0)	7.0 (5.0–11.8)	7.0 (4.0–11.0)	0.344
Smoking, n (%)	90 (24.8)	14 (23.3)	76 (25.1)	0.774
Alcoholism, n (%)	30 (8.3)	6 (10.0)	24 (7.9)	0.608
Body temperature, median (IQR), °C	36.6 (36.4–36.9)	36.6 (36.4–37.0)	36.6 (36.4–36.8)	0.335
Heart rate, median (IQR), bpm	86.0 (78.0–99.0)	90.0 (80.0–110.0)	85.0 (77.0–97.0)	**0.004**
Respiratory rate, median (IQR), bpm	20.0 (18.0–20.0)	20.0 (18.0–20.8)	20 (18.0–20.0)	0.103
Diabetes mellitus, n (%)	94 (25.9)	26 (43.3)	68 (22.4)	**0.001**
Hypertension, n (%)	51 (14.0)	14 (23.3)	37 (12.2)	**0.024**
Multispace involvement, n (%)	168 (46.3)	55 (91.7)	113 (37.3)	**< 0.001**
Gas formation, n (%)	99 (27.3)	43 (71.7)	56 (18.5)	**< 0.001**
Initial onset of symptoms and signs				
Pharyngalgia, n (%)	241 (66.4)	46 (76.7)	195 (64.4)	0.065
Odynophagia, n (%)	198 (54.5)	40 (66.7)	158 (52.1)	**0.039**
Neck pain, n (%)	239 (65.8)	38 (63.3)	201 (66.3)	0.654
Neck swelling, n (%)	216 (59.5)	36 (60.0)	180 (59.4)	0.932
Dysphagia, n (%)	133 (36.6)	34 (56.7)	99 (32.7)	**< 0.001**
Trismus, n (%)	78 (21.5)	13 (21.7)	65 (21.5)	0.971
Hoarseness, n (%)	23 (6.3)	9 (15.0)	14 (4.6)	**0.006**
Dyspnea, n (%)	45 (12.4)	25 (41.7)	20 (6.6)	**< 0.001**
Primary regions of infection				
Suprahyoid region, n (%)	248 (68.3)	46 (76.7)	202 (66.7)	0.128
Infrahyoid region, n (%)	78 (21.5)	1 (1.7)	77 (25.4)	**< 0.001**
Retropharyngeal region, n (%)	37 (10.2)	13 (21.7)	24 (7.9)	**0.001**
Laboratory test				
NEUT, median (IQR), %	80.9 (72.9–86.5)	87.0 (84.2–90.5)	78.9 (71.3–84.9)	**< 0.001**
PLR, median (IQR)	179.1 (132.3–244.7)	232.5 (182.2–351.5)	171.8 (124.0–226.7)	**< 0.001**
RBC, median (IQR), 10^12^/L	4.6 (4.2–5.0)	4.5 (4.1–4.9)	4.6 (4.3–5.0)	0.143
Hb, median (IQR), g/L	135.0 (124.0–146.0)	133.0 (117.0–145.8)	136.0 (126.0–146.0)	0.362
Blood glucose, median (IQR), mmol/L	6.0 (5.1–9.2)	8.3 (6.1–13.5)	5.8 (5.0–8.0)	**< 0.001**
Albumin, median (IQR), g/L	38.4 (33.8–42.6)	31.9 (27.0–36.2)	39.1 (35.9–43.1)	**< 0.001**

Bold p values are statistically significant

*DNSA* deep neck space abscess, *IQR* interquartile range, *bpm* beat per minute, *NEUT%* percentage of neutrophile, *PLR* platelet count to lymphocyte count ratio, *RBC* red blood cell, *Hb* hemoglobin

**Fig. 2:**
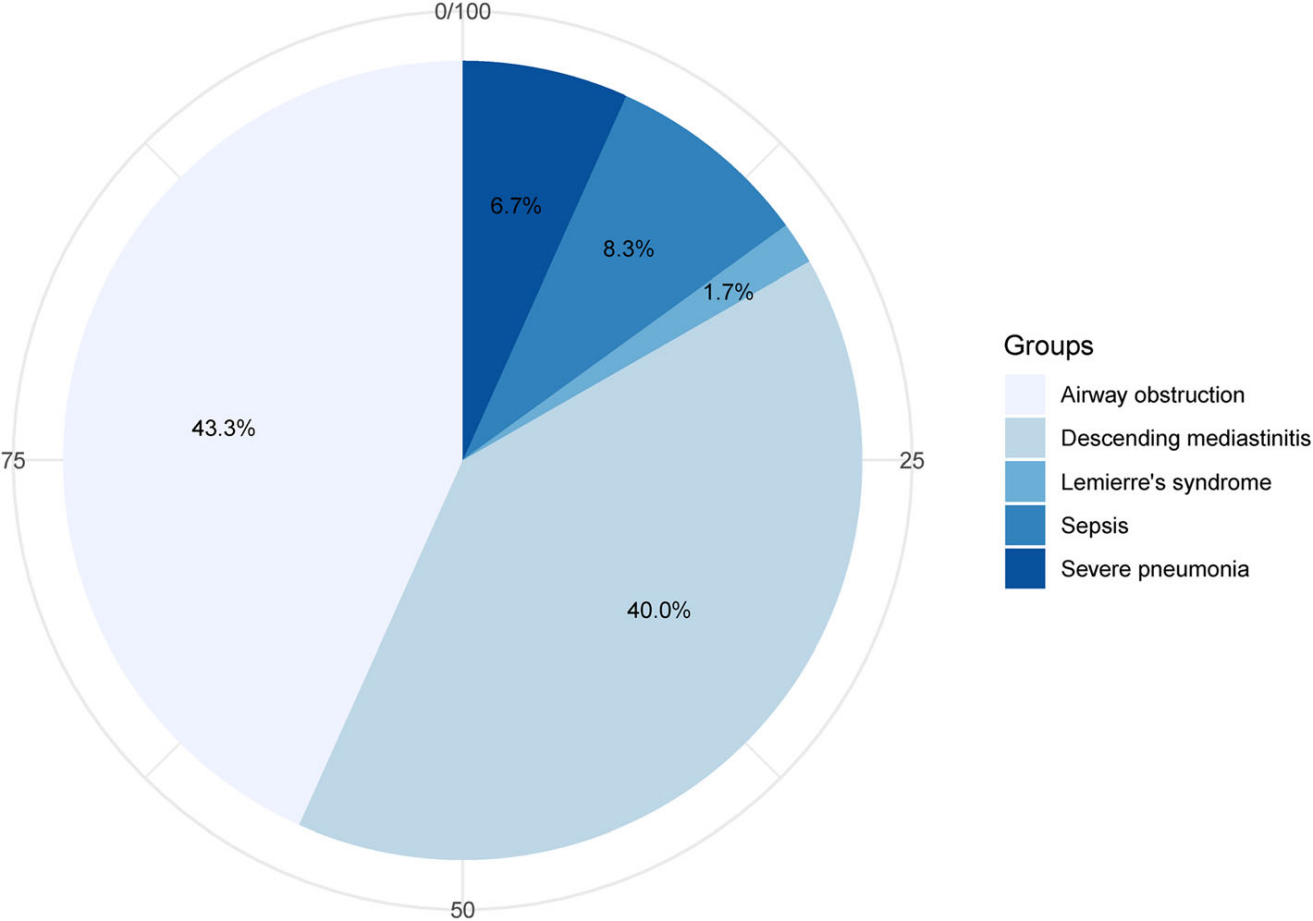
Life-threatening complications in patients who required airway management

The overall mortality rate was 1.1%, but none of the patients died of acute respiratory distress. The causes of death were Lemierre syndrome (1/363; 0.3%), septic shock (2/363; 0.6%), and multiple organ failure (1/363; 0.3%). In total, 125 (34.4%) patients required general anesthesia for surgical drainage in the non-airway management group, 111 (88.8%) of whom were extubated immediately after the operation, while the remaining (14/125, 11.2%) patients were extubated within 24 h after the operation. Sixty patients (16.5%) required airway management (endotracheal intubation or tracheostomy) due to respiratory distress, 55 (91.7%) of whom required subsequent surgical drainage. Of these patients, 29 (48.3%) underwent tracheostomy and 31 (51.7%) underwent endotracheal intubation. The median duration of endotracheal intubation was 3 days (IQR 2–9 days). Patients who required airway management had the following concomitant life-threatening complications: airway obstruction (26/60, 43.3%), descending mediastinitis (24/60, 40.0%), sepsis (5/60, 8.3%), severe pneumonia (4/60, 6.7%), and Lemierre’s syndrome (1/60, 1.7%) (Fig. 2).

### Predictor screen

**Fig. 3:**
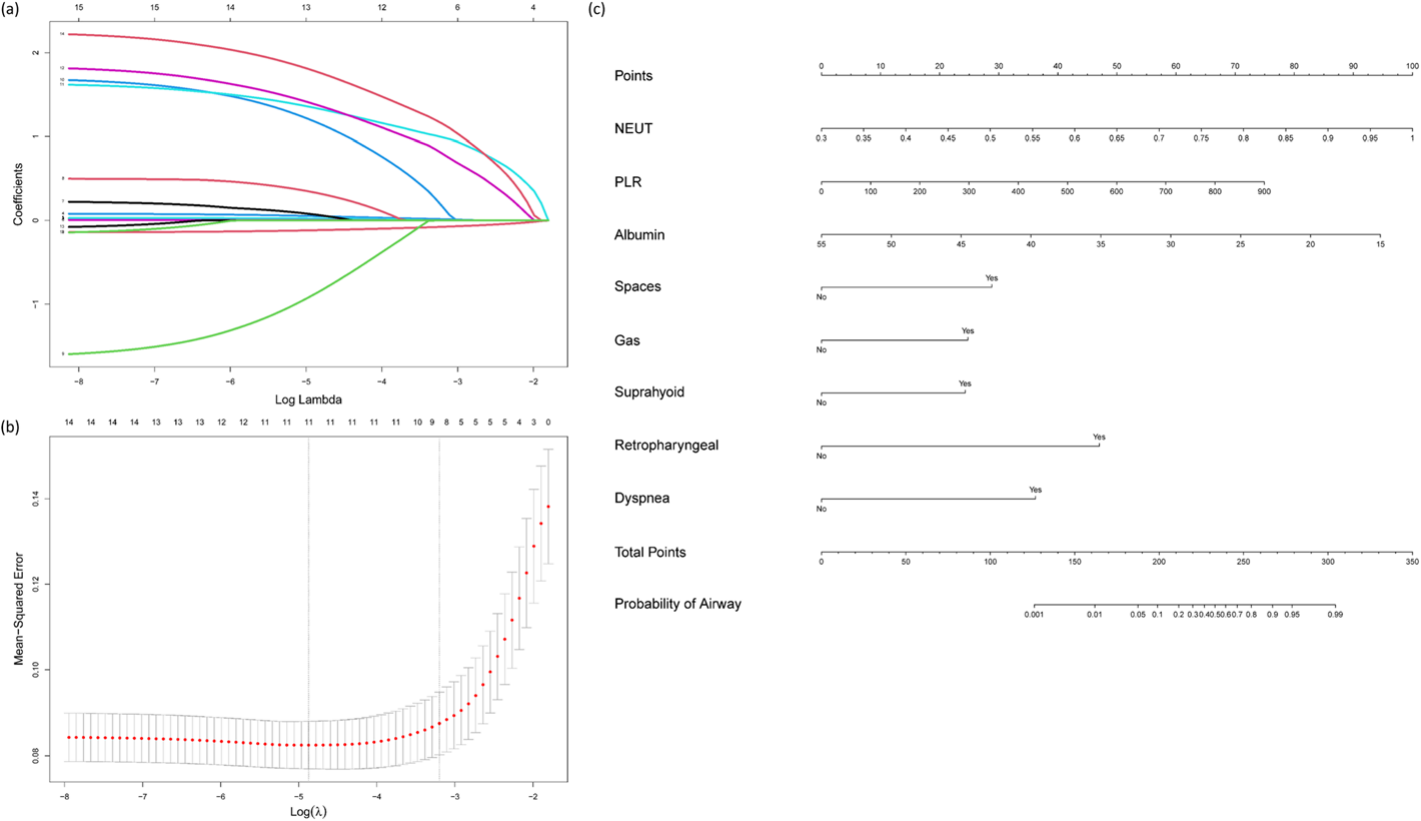
LASSO and logistic regression models on selecting variables. **a** LASSO coefficient profiles of 16 clinical features. **b** Identification of the optimal penalization coefficient λ (0.041) in the LASSO regression model with 10-fold cross-validation and 1 se criterion. **c** Nomogram for assessing the risk of requiring airway management in patients with DNSA. LASSO, least absolute shrinkage and selection operator; DNSA, deep neck space abscess; NEUT, percentage of neutrophile; PLR, platelet count to lymphocyte count ratio; Gas, gas formation; Spaces, multispace involvement

A total of 30 features were collected from each patient in the training cohort. After excluding irrelevant and redundant features that were not statistically significant in univariate analysis (Table 1), 16 features remained for LASSO regression analysis. The results showed that 7 features were strongly associated with airway management when the optimal λ value was 0.041 (Fig. 3a, b). We then built a prediction model using logistic regression and plotted a risk score based on these 7 features (*p* < 0.05): multispace involvement (OR 6.42, 95% CI 1.79–23.07, *p* < 0.001), gas formation (OR 4.95, 95% CI 2.04–12.00, *p* < 0.001), dyspnea (OR 10.35, 95% CI 3.47–30.89, *p* < 0.001), primary region of infection, NEUT% (OR 1.10, 95% CI 1.02–1.18, *p* = 0.015), PLR (OR 1.01, 95% CI 1.00–1.01, *p* = 0.010), and albumin (OR 0.86, 95% CI 0.80–0.92, *p* < 0.001) (Table 2).

**Table 2:** Multivariate analysis of airway management for patients with DNSA in the training cohort

Predictors	OR (95% CI)	p
Multispace involvement	6.42 (1.79–23.07)	0.004
Gas formation	4.95 (2.04–12.00)	< 0.001
Dyspnea	10.35 (3.47–30.89)	< 0.001
Regions of infection		
Infrahyoid	Reference	
Suprahyoid	4.80 (0.53–43.30)	0.162
Retropharyngeal	20.75 (2.04–211.35)	0.010
NEUT%	1.10 (1.02–1.18)	0.015
PLR	1.01 (1.00–1.01)	0.010
Albumin	0.86 (0.80–0.92)	< 0.001

*DNSA* deep neck space abscess, *NEUT%* percentage of neutrophile, *PLR* platelet count to lymphocyte count ratio, *RBC* red blood cell, *Hb* hemoglobin, *OR* odds ratio, *CI* confidence interval

### The performance of the risk score

An online calculator (https://7-414-5-19.shinyapps.io/ClinicalRiskScore/) based on the risk score (Fig. 3c) was developed to allow clinicians to enter the values of the 7 variables required for the risk score with automatic calculation of the likelihood (with 95% CIs) that a patient with DNSA will need airway management.

**Fig. 4:**
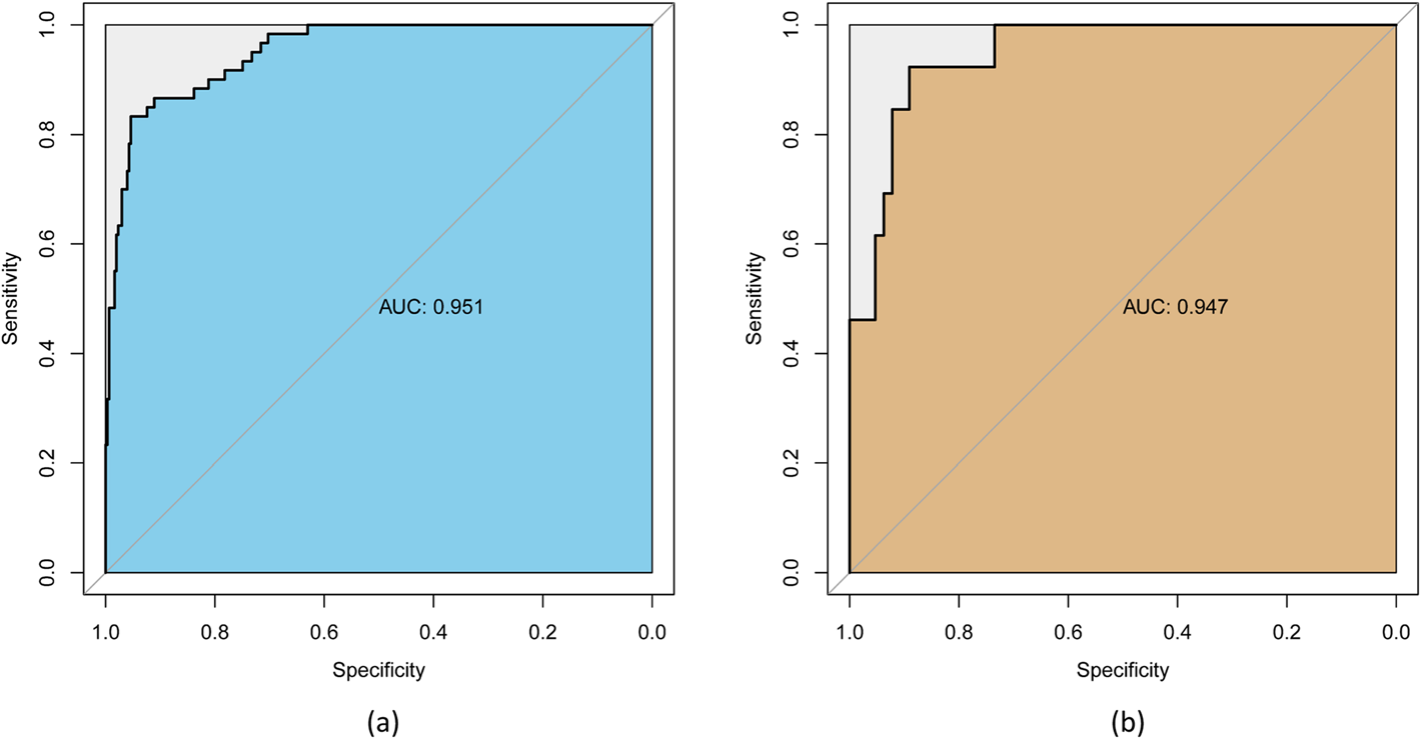
The performance of the ROC curves in the training (**a**) and validation (**b**) cohorts for the risk score. ROC, receiver operating characteristic

**Fig. 5:**
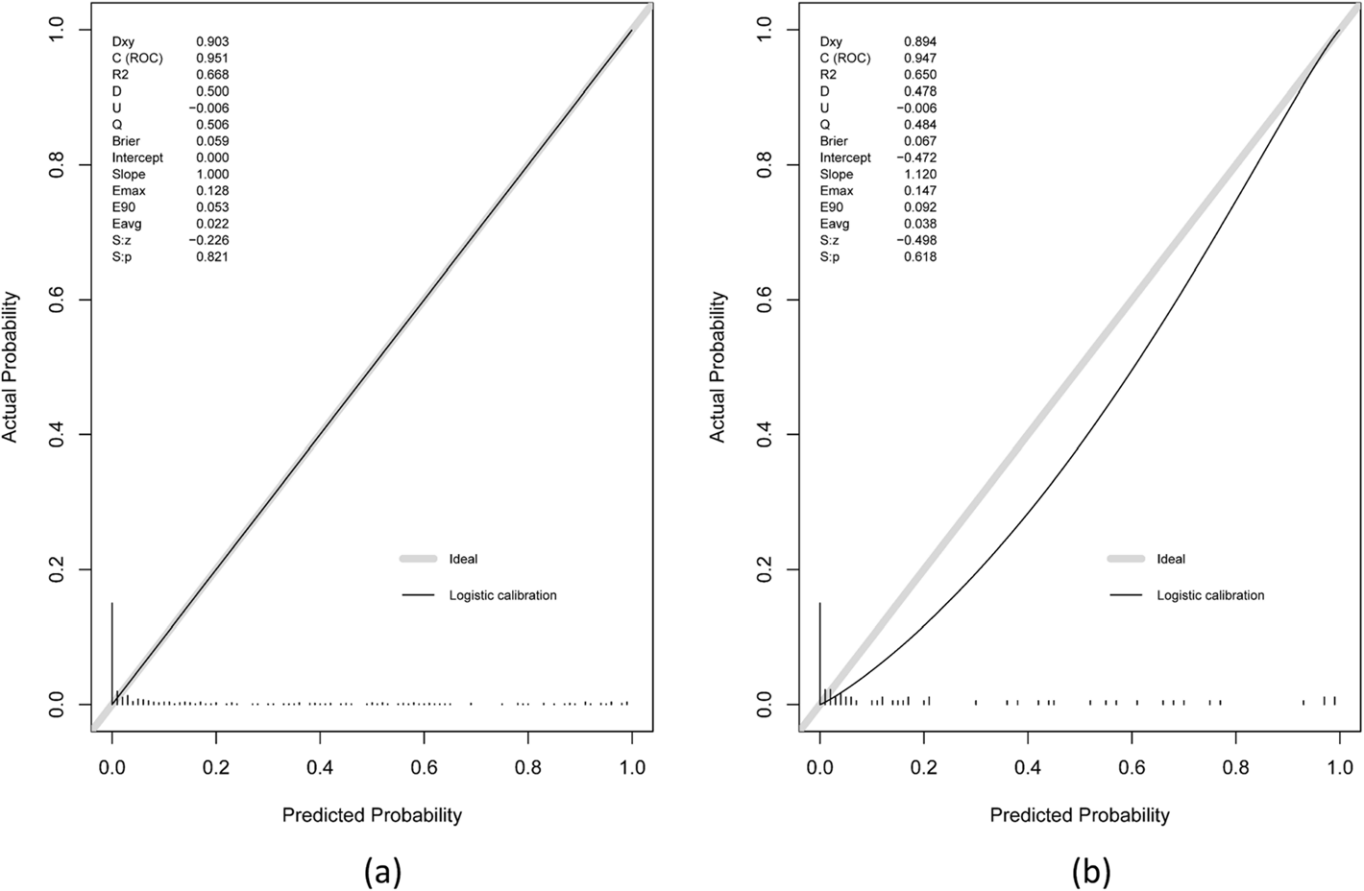
The performance of the calibration plots in the training (**a**) and validation (**b**) cohorts for the risk score

In internal validation, the ROC curve showed that the resulting risk score had good discrimination, with an AUC of 0.951 (95% CI 0.924–0.971; sensitivity 83.3%; specificity 95.4%) (Fig. 4a). The HL test revealed no statistical significance (*p* = 0.821), suggesting a good fit of the model. In addition, the calibration plot graphically showed that the prediction and observation data agreed well in the training cohort (Fig. 5a).

**Fig. 6:**
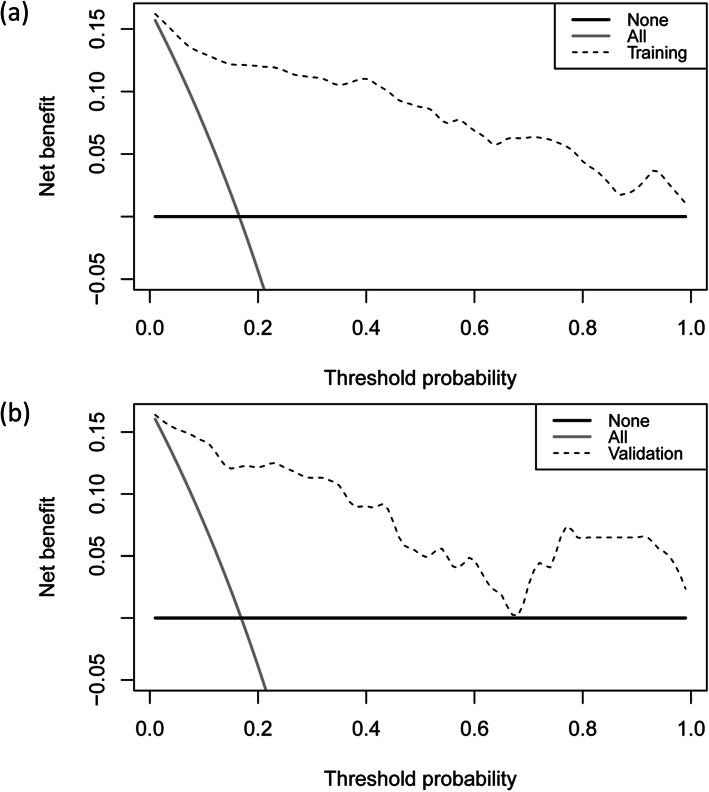
Decision curve analyses in the training (**a**) and validation cohorts (**b**) for the risk score

The cutoff point of the total points was 226.7 (corresponding to a threshold probability of 39.8%). Moreover, the DCA of the risk score in the training cohort is shown in Fig. 6a. With any threshold probability, using this risk score to identify patients who might require airway management would be advantageous over the “treat-all-patients” or “treat-none” schemes.

### The external validation of the risk score

The validation cohort (62 males and 15 females) included 77 patients from 6 hospitals between January 1, 2020, and December 31, 2020. The median age of patients in the validation cohort was 51.0 years (IQR 39.7–56.5 years). Thirteen patients (16.9%) required airway management. Of these patients, 5 (38.5%) underwent tracheostomy, and 8 (61.5%) underwent endotracheal intubation. The median duration of intubation was 5 days (IQR 2.3–12.8 days). Variables used in the risk score for airway management in the validation cohort are shown in Table 3. In this external validation, the risk score displayed good discrimination, with an AUC of 0.947 (95% CI 0.871–0.985, sensitivity 92.3%, specificity 89.1%) (Fig. 4b). Good calibration was also demonstrated by a nonstatistical significance obtained in the HL test (*p* = 0.618), as displayed by the calibration curve (Fig. 5b). DCA showed that the decision-making for airway management still had good benefits for patients (Fig. 6b).

**Table 3:** Demographics and clinical characteristics of patients with DNSA in the validation cohort

Predictors	All (n = 77)	Airway management		p
		Yes (n = 13)	No (n = 64)	
Age, median, (IQR), years	51.0 (39.7–56.5)	48.0 (40.5–56.5)	51.0 (39.0–56.8)	0.965
Sex, male, n (%)	62 (80.5)	11 (84.6)	51 (79.7)	1.000
Multispace involvement, n (%)	45 (58.4)	13 (100.0)	32 (50.0)	< 0.001
Gas formation, n (%)	25 (32.5)	9 (69.2)	16 (25.0)	0.003
Dyspnea, n (%)	17 (22.1)	8 (61.5)	9 (14.1)	0.001
Primary regions of infection				
Suprahyoid region, n (%)	52 (67.5)	12 (92.3)	40 (62.5)	0.050
Infrahyoid region, n (%)	17 (22.1)	0 (0.0)	17 (26.6)	0.060
Retropharyngeal region, n (%)	8 (10.4)	1 (7.7)	7 (10.9)	0.728
Laboratory test				
NEUT, median, (IQR), %	84.1 (76.1–87.6)	87.5 (84.7–91.1)	82.3 (75.0–86.4)	0.008
PLR, median, (IQR)	189.3 (132.7–287.6)	320.7 (128.8–410.2)	172.5 (132.3–261.5)	0.028
Albumin, median, (IQR), g/L	38.0 (32.9–42.2)	29.0 (26.9–34.9)	39.7 (34.6–42.7)	< 0.001

*DNSA* deep neck space abscess, *IQR* interquartile range, *NEUT%* percentage of neutrophile, *PLR* platelet count to lymphocyte count ratio

## Discussion

In this study, the clinical risk score consisted of 7 features that were independent predictors for airway management in patients with DNSA, including multispace involvement, gas formation, dyspnea, primary region of infection, NEUT%, PLR, and albumin. The performance of this risk score demonstrated good discrimination and calibration as assessed by AUC values, HL tests, and calibration plots. In addition, DCAs further indicated that our prediction model conferred significantly high clinical net benefit in the training and validation cohorts, which was of great value for an accurate individualized assessment of the requirement for airway management in patients with DNSA.

Dyspnea is an important predictor for airway management in patients with DNSA, but it is a rare clinical feature with an incidence of 5.6–12.8% at hospital visits [3–5], which is roughly the same (12.5%) as our study (Table 1). We also found that 58.6% of patients who required airway management did not have obvious dyspnea at the hospital visit, which may tend to underestimate the progression of the patient’s condition. Thus, relying only on dyspnea to assess whether patients require airway management lacks the support of evidence, so we need to identify novel features from clinical data to help illustrate the necessity of airway management in patients with DNSA.

Independent predictors in the risk score, such as NEUT%, albumin level, multispace involvement, and gas formation, have been reported to be significantly associated with poor condition in DNSA [10, 14, 15, 18]. PLR is a novel inflammatory indicator that can reflect disease severity in infectious diseases [16, 17]. Furthermore, these predictors can be simply and easily obtained from physical examination, laboratory tests, and contrast-enhanced CT scanning. These tests are routine and essential in patients with DNSA, so we can quickly calculate the risk score based on this information and make clinical decisions in a timely manner.

Based on the hyoid bone, we divided the primary infection into 3 regions: suprahyoid, infrahyoid, and retropharyngeal regions. Ludwig’s angina is one of the serious complications occurring in the suprahyoid region [20]. Infection can spread rapidly in a short time period to multiple deep neck spaces, resulting in trismus and dyspnea [21]. Primary infection in the retropharyngeal region is mainly caused by trauma, such as foreign body injury [22], which aligns with the findings of our study (20/37; 54.1%). Foreign bodies can not only damage the retropharyngeal mucosa but also reach the retropharyngeal, danger, and prevertebral spaces [23]. If infection occurs, it can not only cause airway compromise but also spread downward rapidly, resulting in descending mediastinitis and pericardial abscess. This downward infection can also result from the suprahyoid region. Furthermore, pus aspiration can occur in patients with retropharyngeal abscesses, as the pus can flow directly into the airway through a mucosal fistula. All of these conditions have a higher risk of airway loss and require airway management.

Interestingly, even if an infection involves multiple spaces and is large, it may still not complicate airway management if it is located in an area such as the carotid, pretracheal, and paratracheal space, which might appear serious externally [24]. We also found that patients with a primary infection in the infrahyoid region were unlikely to require airway management.

Thiago et al. [5] reported that 16.8% of their patients required airway management, which is similar to the proportion in our study (16.6%). Although the sample size of patients who needed airway management is small, possibility limiting the generalizability of the risk score, the results of the ROC curves, HL tests, and calibration plots in the training and the validation cohorts show that the generalizability of the risk score is acceptable (Figs. 4 and 5). Moreover, the DCAs of the risk score in the training and the validation cohorts also confirmed that the risk score could benefit the patients, and overtreatment was not observed (Fig. 6).

We suggested categorizing the patients into low- and high-risk groups based on the cutoff points of 226.7 (39.8%), which was calculated by maximizing the Youden index in the training cohort. The sensitivity and specificity of the model was 83.3% and 95.4%, respectively. Although the predictive threshold of 39.8% is not high, it can suggest the need for vigilance to clinicians with regard to patients who are at high risk of needing airway management. Preparations for airway management (tracheostomy or endotracheal intubation), such as placing a tracheostomy instrument set beside the bed, contacting anesthesiologists to obtain a consultation with regard to the evaluation of airway management procedures, configuring ventilators, ensuring adequate doctor-patient communication, and increasing nursing care, should be initiated for patients in the high-risk group. Such preparations can ensure that any subsequent airway management is performed according to the plan, thereby reducing the urgency of the procedure, increasing the efficiency of medical resource allocation, and avoiding acute hypoxia. If endotracheal intubation is successfully performed, we recommend retaining the endotracheal tube and admitting the patient to the ICU to facilitate mechanical ventilation. For low-risk patients, a “watch and wait” approach could be employed with the use of empiric antibiotics, and drainage surgery as necessary.

This is the first clinical risk score for airway management of DNSAs, and it exhibited good performance in the training and validation cohorts. However, there are still several limitations. First, many potential predictors were not included in the construction of this clinical risk score because of the large number of missing values, even though they may be closely related to airway management, such as limited movement of the neck, C-reactive protein, and procalcitonin. The results may be influenced as these potential predictors are available. Second, most of the patients received drug intervention by themselves or in other medical institutions before the hospital visit, and some predictors may be affected by this situation, such as NEUT%, PLR, and albumin. The score may be biased for patients without any drug intervention.

DNSA is a relatively rare disease, which may lead to the uneven distribution of patients among centers, some of which have limited resources. For instance, some centers mainly receive critically ill patients, while some centers mainly receive patients in less critical condition. Using data from centers that usually treat critically ill patients to validate a score developed based on data from centers that usually treat less critically ill patients would produce inaccurate results. We attempted to avoid this problem by pooling data from multiple clinical centers in the Pearl River Delta region of Guangdong Province. However, our data may not be nationally representative. Living habits and cultural customs differ between southern and northern regions of China. There are also substantial differences between these regions in the incidence of DNSA in populations with specific diseases, such as diabetes and hypertension. Thus, the risk score may not be accurate in other regions of the country. However, we hope to conduct a prospective, national, multicenter study in the future based on our model and to develop regional personalized prediction models and clinical guidelines for airway management in DNSA patients.

## Conclusions

The risk score demonstrated good diagnostic ability and calibration in predicting the requirement of airway management in patients with DNSA. Meanwhile, it has also been converted into an online calculator for clinicians to use simply and friendly. The risk score could help early identify whether a patient requires airway management, thus facilitating multidisciplinary cooperation, advancing patient safety, and supporting appropriate treatment.

## Data Availability

The datasets used and/or analyzed during the current study are available from the corresponding author on reasonable request.

## Abbreviations

DNSA: Deep neck space abscess
ICD-10-CM: International Classification of Diseases, Tenth Revision, Clinical Modifications
LASSO: Least absolute shrinkage and selection operator
AUC: Area under the curve
CI: Confidence interval
HL: Hosmer–Lemeshow
CT: Computed tomography
ED: Emergency room
NEUT%: Percentage of neutrophils
PLR: Platelet count to lymphocyte count ratio
RBC: Red blood cell
Hb: Hemoglobin
ASA: American Society of Anesthesiologists
ICU: Intensive care unit
IQR: Interquartile range
OR: Odds ratio
ROC: Receiver operating characteristic
DCA: Decision curve analysis
